# Computer-Aided System for the Detection of Multicategory Pulmonary Tuberculosis in Radiographs

**DOI:** 10.1155/2020/9205082

**Published:** 2020-08-24

**Authors:** Yilin Xie, Zhuoyue Wu, Xin Han, Hongyu Wang, Yifan Wu, Lei Cui, Jun Feng, Zhaohui Zhu, Zhongyuanlong Chen

**Affiliations:** ^1^Department of Information Science and Technology, Northwest University, Xi'an, Shaanxi 710127, China; ^2^School of Computer Science and Technology, Xi'an University of Posts and Telecommunications, Xi'an, Shaanxi 710121, China; ^3^Shaanxi Key Laboratory of Network Data Analysis and Intelligent Processing, Xi'an University of Posts and Telecommunications, Xi'an, Shaanxi 710121, China; ^4^State-Province Joint Engineering and Research Center of Advanced Networking and Intelligent Information Services, School of Information Science and Technology, Northwest University, Xi'an 710127, Shaanxi, China; ^5^Chest Hospital of Xinjiang Uyghur Autonomous Region of the PRC, Urumqi, Xinjiang Uygur Autonomous Region 830049, China

## Abstract

The early screening and diagnosis of tuberculosis plays an important role in the control and treatment of tuberculosis infections. In this paper, an integrated computer-aided system based on deep learning is proposed for the detection of multiple categories of tuberculosis lesions in chest radiographs. In this system, the fully convolutional neural network method is used to segment the lung area from the entire chest radiograph for pulmonary tuberculosis detection. Different from the previous analysis of the whole chest radiograph, we focus on the specific tuberculosis lesion areas for the analysis and propose the first multicategory tuberculosis lesion detection method. In it, a learning scalable pyramid structure is introduced into the Faster Region-based Convolutional Network (Faster RCNN), which effectively improves the detection of small-area lesions, mines indistinguishable samples during the training process, and uses reinforcement learning to reduce the detection of false-positive lesions. To compare our method with the current tuberculosis detection system, we propose a classification rule for whole chest X-rays using a multicategory tuberculosis lesion detection model and achieve good performance on two public datasets (Montgomery: AUC = 0.977 and accuracy = 0.926; Shenzhen: AUC = 0.941 and accuracy = 0.902). Our proposed computer-aided system is superior to current systems that can be used to assist radiologists in diagnoses and public health providers in screening for tuberculosis in areas where tuberculosis is endemic.

## 1. Introduction

Tuberculosis is a communicable disease that is one of the top 10 causes of death worldwide. At least 10 million people were estimated to have contracted tuberculosis in 2018 [1]. However, with a timely diagnosis and treatment with first-line antibiotics, most people who develop tuberculosis can be cured, and their transmission of the infection can be curtailed. Chest radiography [2], mycobacterium tuberculosis cultures [3], and sputum smear microscopy [4, 5] are commonly used methods to diagnose tuberculosis. Since nearly 86% percent of the tuberculosis cases in the WHO regions were low- and middle-income countries in 2018 [1], the chest radiography (CXR or chest X-ray) is an important tool for tuberculosis screening and provides useful help in diagnosing tuberculosis since it is a noninvasive, low radiation dose, reduced cost, readily available procedure that can conduct accurate detection. However, a major limitation of chest radiography is that it requires experienced radiologists, and few experienced radiologists are available in these countries. Thus, some computer-aided systems have been developed in recent years that use digital techniques to detect the physiological and pathological conditions of various diseases, including breast cancer [6] and tuberculosis. Many commercial products have been developed for clinical use, including the CAD4 TB, Riverrain, and Delft imaging systems [7]. However, due to the complexity of chest radiographs, the automatic detection of the disease remains unresolved, and most existing computer-aided systems are designed to detect lung cancer. Relatively few studies have focused on the automatic detection of other pathologies [8], such as tuberculosis. The current computer-aided systems for tuberculosis use the following three research methods for chest radiographies.

The most common method estimates whether CXRs contain tuberculosis by classifying the chest X-ray images. Binu Joykutty et al. [9] used adaptive thresholds to segment chest images with different illuminances, extracted the feature information contained in the images, and used the k-nearest neighbours classifier to classify chest images. Ginneken et al. [10] aimed to detect signs of diffuse texture abnormalities in chest radiographs to determine chest radiograph abnormalities, which is similar to [11]. Govindarajan et al. [12] used Bag of Features approach with Speeded-Up Robust Feature descriptor to classify TB CXRs. These existing algorithms use well-designed morphological features to improve screening performance, and the main process includes image preprocessing, feature extraction, and feature classification. However, manually denoted features cannot guarantee the best tuberculosis classification.

The next type of method detects and estimates whether a certain lesion in a CXR is tuberculosis. Shen et al. [13] adopted a hybrid knowledge-based Bayesian classification method to automatically detect tuberculosis cavities. Xu et al. [14] also researched the detection of cavities. Maduskar et al. [15] proposed a method to locate ribs using the chest wall contour as the landmark to automatically detect pleural effusion. These systems use only one type of tuberculosis lesion for target detection, and thus they cannot achieve comprehensive tuberculosis screening. However, tuberculosis has multiple causes, and each cause exhibits different symptoms of the disease.

Recently, some studies based on deep learning models [16] have offered good alternatives to other conventional classification methods. Lopes et al. [17] first proposed a method that combines pretrained CNNs and a Multiple Instance Learning algorithm to maximize the ability of CNNs to identify different types of pathologies in CXRs. Sivaramakrishnan et al. [18] evaluated the performance of a CNN-based deep learning model for tuberculosis screening using CXRs, which is similar to [19]. Hwang et al. [20] used transfer learning to improve the TB screening performance using convolutional neural networks. Mohammad et al. [21] used a deep convolution shallow network to extract features and improve the detection accuracy. These methods avoid the problem of manually extracting features by automatically extracting deep high-level hierarchical features for tuberculosis classification directly from the input raw data. However, these systems use whole chest radiographs as classification targets. This results in the disadvantage of low effective information density. In the feature extraction process, it is also not easy to directly extract the most effective features from the input information, which leads to misclassifications due to unclear decision boundaries.

**Figure 1:**
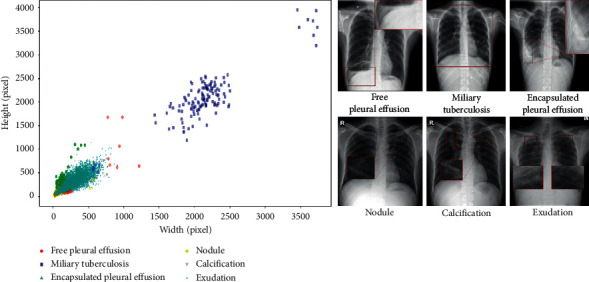
The distributions and sizes of the six tuberculosis lesions, with the width as the abscissa and the height as the ordinate.

**Figure 2:**
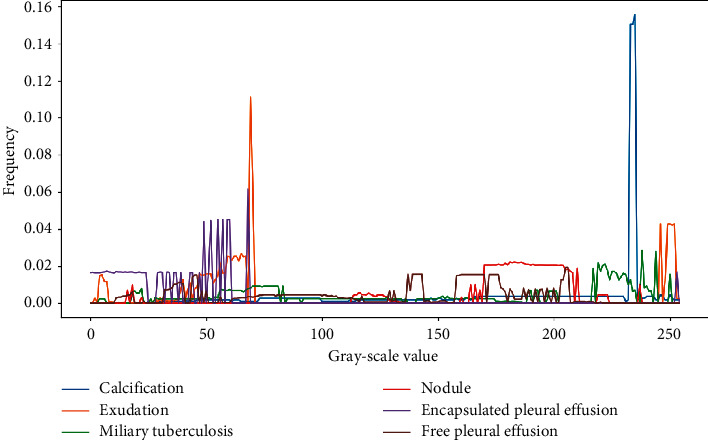
The relationship between the frequency of occurrence of each grey-scale value and the grey-scale value in the tuberculosis lesion image, with the grey-scale value as the abscissa and the frequency as the ordinate.

These studies are indispensable explorations for the establishment of X-ray-based tuberculosis computer-aided systems, but there is space for improvement in practical applications. We provided a tuberculosis dataset labelled by two professional radiologists, which covered almost all types of tuberculosis lesions, including exudation, nodules, calcification, miliary tuberculosis, encapsulated pleural effusion, and free pleural effusion. As shown in Figure 1, we analyze the distributions and sizes of these six lesions and find that the lesion areas cover a wide range. Among them, the lesion area of miliary tuberculosis is generally dispersed over the whole lung, and lung nodules occupy only a small area in the lung. These lesions have different sizes and positions in chest radiography. In particular, small lesions account for most of the legions in the entire lesion detection task. As shown in Figure 2, we plot the probability distribution map corresponding to each grey-scale level of the image of the lesion area for analysis and analyze the significant differences between the lesions. We find that some lesions have similar characteristics and are difficult to distinguish. These problems pose challenges for detecting the multiple categories of tuberculosis lesions.

Therefore, this paper is different from the previous pulmonary tuberculosis analysis methods. Instead of using the whole image as a classification target, we focus on the specific tuberculosis area for analysis. We apply an effective lesion detection method to the image, which reduces the amount of calculations and also allows us to obtain more accurate analysis results. Meanwhile, in order to avoid the problem of manually extracting image features, a computer-aided system based on deep learning is proposed for the detection of multiple categories of tuberculosis lesions in radiographs. Our proposed system can effectively solve these challenges, and it includes a two-stage process for the accurate detection of tuberculosis.

The first stage in the design of the chest radiographic computer-aided system is the correct segmentation of the lung region from a CXR [22]. In computer-aided system, the detection and segmentation of lesions are very important [23]. So, in the proposed system, we consider the problem that patients with lung diseases have reduced contrast between their lung fields and boundaries [24], which poses a challenge to the segmentation task. U-net [25] was used to perform lung segmentation for CXR images since it can perform end-to-end training using very few images and handle complex shape changes and the surrounding tissue. The segmentation stage has achieved good results on two public datasets [26] and outperforms the previous segmentation methods using different convolutional neural network structures [27–31]. The segmentation of lung fields is a prerequisite step to precisely define a region-of-interest and is subsequently used in the detection stage of the computer-aided system.

In the second stage, this paper proposes a multicategory tuberculosis lesion detection scheme for the first time, which is different from the methods that classify the whole CXR image and the previous single-category symptom detection methods. Various object detection methods using deep learning have been reported in the computer vision literature [32–34]. The region-based convolutional network (RCNN) aims to obtain the bounding boxes of the targets using location and size information. Unfortunately, the RCNN is computationally expensive and extremely slow. Therefore, by using the Faster RCNN network [32] as the main framework of our multicategory tuberculosis lesion detection network, we can increase the object detection sensitivity and speed. For the problem that the areas of some lesions in the CXR image are too small to be detected, we introduced a new feature pyramid architecture in the Faster RCNN. The experimental results proved that the proposed method greatly improved the detection accuracy of nodules or calcifications with small areas. Furthermore, the training of the lesion detection models is optimized to reduce the number of false positives.

Finally, in order to further illustrate the effectiveness of the proposed system, we propose a classification rule based on the tuberculosis lesion detection model. We compare the performance of the existing tuberculosis classification methods based on a convolutional neural network in a computer-aided system. We have achieved good performance on two public datasets [26], which exceeds the performances of existing methods. The proposed system can effectively improve the diagnostic efficiency of radiologists for tuberculosis and can be used to effectively screen tuberculosis by public health organizations.

## 2. Materials and Methods

**Figure 3:**
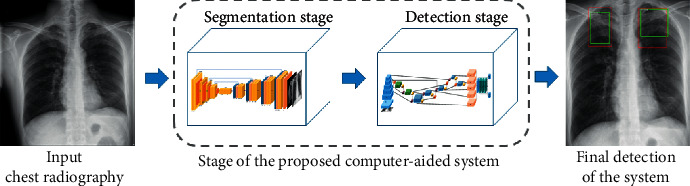
Comprehensive view of the computer-aided system for tuberculosis diagnosis using chest radiography.

In this study, we propose a computer-aided system based on deep learning for detecting multiple categories of tuberculosis lesions in radiographs. A schematic diagram of the proposed computer-aided system is illustrated in Figure 3. A fully convolutional neural network is used for the automatic lung segmentation from Chest X-rays. Then, the segmented chest X-ray images with multilabel lesions are used to train the object detection network with the Faster RCNN that introduces the learning scalable feature pyramid architecture and obtain the object detection model of the lesion area to determine the location and category of the tuberculosis.

### 2.1. Dataset

The JSRT dataset was gathered by the Japanese Society of Radiological Technology. It consists of 247 CXRs. 93 are normal and 154 are abnormal with diﬀerent TB manifestations. Each image in the dataset is stored in PNG format with 2048 × 2048 pixels and has a grey-scale depth of 12 bits. The lung masks of the images are provided by the same authors in another dataset named the SCR dataset [35]. So, the JSRT dataset was used to segment the lung regions.

The Montgomery dataset was created by the U.S. National Library of Medicine in collaboration with the Department of Health and Human Services, Montgomery County, Maryland, USA. It contains 80 CXRs that have been classiﬁed as normal by doctors and 58 CXRs that have TB manifestations. The images are stored in PNG format with a pixel resolution of 4020 × 892 pixels with a grey-scale depth of 12 bits. The corresponding manual lung masks that were generated under the supervision of radiologists are also available. The Shenzhen dataset was collected in the Guangdong Hospital in Shenzhen, China. The dataset contains 662 frontal CRs. 336 of those have tuberculosis inflections, and 326 are normal. All image resolutions are approximately 3,000 × 3,000 pixels [26]. Therefore, the Montgomery and JSRT datasets were used to detect tuberculosis; besides, the Montgomery dataset also was used for the segmentation of lung.

The local dataset was collected from the First Affiliated Hospital of Xi'an Jiao Tong University (FAHXJU) in Shanxi, China. The FAHXJU dataset contained 2382 CXRs classified as normal and 2962 CXRs classified as containing tuberculosis by two professional physicians. The resolution of the images ranges from 858 × 1004 to 3480 × 4240 pixels. The local dataset was collected to detect multiple categories of tuberculosis signs. The most common seen signs of tuberculosis, including exudation, nodules, calcification, miliary tuberculosis, encapsulated pleural effusion, and free pleural effusion, were defined according to the glossary of terms for thoracic imaging of Fleischner Society [36]. Since the annotations were intended for computerized pattern recognition, the regions of interests have to delineate very precisely. After learning the glossary, 2962 CR images were randomly selected, and the ground truth of the six signs was annotated by two radiologists double-blindly as a preliminary experiment to test the interobserver consistency for the identification of the six CT signs. The disagreements were decided by a higher experienced radiologist. The physicians used a professional image annotation software called labelimg [37] to label tuberculosis signs. The signs were labelled with rectangle boxes that were as small as possible to decrease the possibility of introducing noises in the training process. Then, the left CR images were annotated by the two radiologists double-blindly by the same way. Disagreements were decided by a higher experienced radiologist. Then, the ground truths were saved as .xml file.

### 2.2. A Fully Convolutional Neural Network for Lung Segmentation

**Figure 4:**
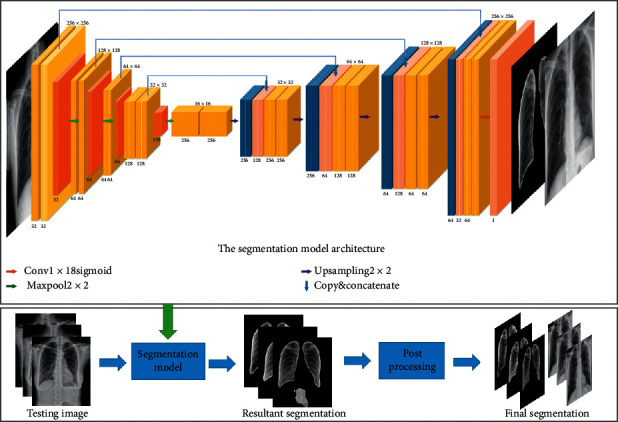
Segmentation model architecture.

Lung segmentation is the primary task in the detection of multicategory tuberculosis lesions in computer-aided systems. In segmentation, it is necessary not only to classify pixels into foreground or background, but also to preserve their spatial information. The previous research has well solved the issues in the use of an encoder-decoder network. Therefore, we chose the fully convolutional neural network U-Net [25] for lung segmentation, and based on the actual situation of the data, a small number of modifications are used, as shown below. The CXRs are resized to a 256 × 256 resolution and then preprocessed for delivery to the U-Net network. As shown in Figure 4, the network structure consists of shrink and expansion modules. After acquiring each image, the shrink module performs two 3 × 3 convolutions (unpadded convolutions). Each convolution is followed by a batch normalization layer and rectified linear unit (ReLU) for the lung segmentation of the full convolutional neural network, and it down samples the image into half its size using the max pooling of 2 × 2. After each iteration, the depth of the generated feature map is doubled, and its spatial size is reduced to half. Then, the expansion module up samples the feature map and connects it with the corresponding feature map in the contraction module; the rest is consistent with the convolution operation of the contraction module [25].

Finally, the trained segmentation network is used for lung segmentation on its own tuberculosis dataset. For the segmented data, there is a small amount of data containing some small holes, small unnecessary objects/points in the lung region or fused left and right lungs. We have postprocessed these data. In the entire computer-aided detection system, the main role of the segmentation stage is to obtain complete lung regions. Therefore, in image postprocessing, the method of image connected region detection is mainly used to detect whether the nonlung region is segmented in the segmented image. Because the nonlung regions in the segmented images are mostly other tissue images with small areas, we use the method of preserving the two largest areas obtained from the image connected area detection, and the small objects are removed by selecting the two foreground objects having the left and right lung regions. In this way, a segmented image of the final lung region is obtained, and then the original chest X-ray is cropped according to the size of the segmented lung region to acquire the data for tuberculosis detection analysis. This method reduces unnecessary tissue images in chest X-rays and avoids the problem of losing some context information due to segmentation. Therefore, the quality of the lung region image sent to the detection stage is not significantly degraded.

### 2.3. A Region-Based Convolutional Network for Multicategory Tuberculosis Lesion Detection

#### 2.3.1. Learning the Scalable Feature Pyramid Architecture of Multicategory Tuberculosis Lesion Detection

This paper focuses on the specific tuberculosis area for analysis. Since the RCNN is computationally expensive and extremely slow, the Faster RCNN network is used as the main framework of the detection stage. The different lesion sizes pose a great challenge to the detection task, especially the detection of small lesions. It is difficult for the traditional detection framework to combine rich semantic information with detailed feature information. The current target detection network uses the feature pyramid network (FPN) structure to solve the multiscale problem, which is very helpful for detecting small objects. It adopts a backbone model that is usually used for image classification. Furthermore, it builds the feature pyramid by sequentially combining two adjacent layers in the backbone model, and the feature pyramid is built via top-down and lateral connections to produce high-resolution and semantically strong feature representations. The FPN is simple and effective, but it may not be the optimal architecture design. Therefore, a better learning scalable feature pyramid structure is used here for the detection of multicategory tuberculosis lesions in chest radiography.

The new FPN architecture is customized using the Neural Architecture Search approach. First, the search space is constituted to cover all cross-scale connections to produce multiscale features. Multiple merging cells constitute the search space, where one merging cell fuses two feature connections from different feature layers to generate the feature output. The feature fusion method here uses nearest neighbour sampling or max pooling to adjust the input feature layer to the output resolution. The merged feature layer will always follow the ReLU, a 3 × 3 convolution, and a batch normalization layer. Then, it uses the reinforcement learning training controller to select the optimal FPN architecture in a given search space. Since the FPN cross-connection is not known, a recurrent neural network is used as a controller and trained using the Near-end Strategy Optimization algorithm to construct different connections. The final FPN structure is obtained via its controller's convergence, and its accuracy is the highest [34].

**Figure 5:**
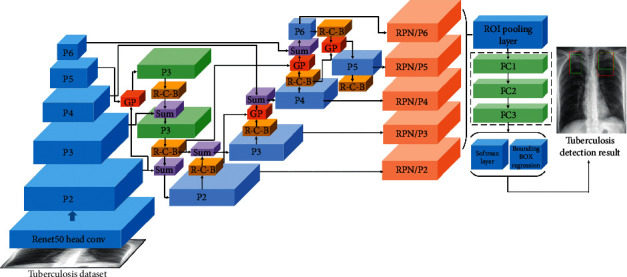
The learning scalable feature pyramid architecture in the Faster RCNN network. The architecture of the discovered 7-merging-cell pyramid network in the NAS-FPN includes 5 input layers (Navy blue) and 5 output feature layers (Light blue). GP and R-C-B stand for Global Pooling and ReLU-Conv-BatchNorm, respectively.

As shown in Figure 5, we apply the learning scalable feature pyramid architecture to the RPN in the Faster RCNN network, which is used to generate the initial candidate area. The specific improvement adjusts the process by replacing the original single-scale feature map of the RPN with the NAS-FPN. The last layer in each set of feature layers is used as the input to the first pyramid network, and the output of the first pyramid network is the input to the next pyramid network. Five scales {C2, C3, C4, C5, and C6} are used as input features, and the corresponding feature steps are {32, 64, 128, 256, and 512} pixels. Then, the input features are passed to the learning scalable feature pyramid architecture, and the pyramid network then outputs the enhanced multiscale features representing {P2, P3, P4, P5, and P6}. We connect the operations of performing class-specific classifiers and the bounding box regression (3 × 3 transformation and 2 sibling 1 × 1 transformations) to each level on the feature pyramid and use multiple anchors with aspect ratios of {1 : 2, 1 : 1, and 2 : 1} at each level, and the rest of the network is consistent with Faster RCNN [32, 33]. It can solve the problem that tuberculosis lesions have different categories, sizes, and positions in chest radiographs, especially that small lesions are not easy to detect.

#### 2.3.2. False-Positive Tuberculosis Lesion Reduction Module

The screening of tuberculosis diseases, especially large-scale screening, is different from daily clinical detection. To find more cases earlier, screening usually emphasizes that the screening technique is more sensitive; therefore, in the proposed computer-aided system, we adjusted the parameters to minimize the detection of false negative lesions, but this resulted in more false positives. This mainly occurs because ordinary lung infections are often similar to tuberculosis in imaging, which makes them difficult to distinguish and prone to misjudgements. Therefore, we added a false-positive tuberculosis lesion reduction module to mine the indistinguishable samples during the detection process. Based on the previous tuberculosis detection model, we send the normal chest X-ray collected by us to the best trained tuberculosis detection module and apply the detector to the normal chest radiograph. After the detection, a portion of the normal chest X-rays will be determined to contain labelled suspected lesion areas. These areas are similar to the appearance of tuberculosis in chest X-rays. Therefore, we apply normal labels for false positive samples. Then, we put them into the negative sample dataset and use reinforcement learning to train the detector again to reduce the detection of false positives.

## 3. Results and Discussion

### 3.1. Experimental Settings

In this work, we use the JSRT [35] and the Montgomery [26] datasets to train and test the segmentation stage. The tuberculosis disease detection stage is trained and tested using the collected tuberculosis dataset. The performance of the proposed computer-aided system in the segmentation and detection phases is gradually evaluated. At each stage, a five-fold cross-validation test is performed using the training and test datasets. These training and testing data are generated via a hierarchical division to ensure that each chest X-ray is tested the same, and any deviation is prevented. In all experiments, 80% of the dataset was used for training and 20% was used for testing.

To train a better deep learning model, we enhance the data by improving the diversity of the training data. In the segmentation stage, we performed related experiments on the size of the input image. Therefore, we uniformly convert the images in the two public datasets [26, 35] to the size of 256 × 256, which is a resolution that satisfactorily retains the structural details of the images, while significantly reducing the model's computational cost. And then rotate and translate these images to expand the size of the training set to 5 times the original size to prevent overfitting the model. In the detection stage, the small lesions account for most of the legions in the entire lesion detection task, so the size of the input cropped chest X-rays in the detection stage cannot be converted too small. At the same time, under the guidance of the experimental performance of reference [34], we convert the local dataset to 1024 × 1024 pixels in consideration of the capacity of GPU memory and detection performance and then randomly adjust the brightness and contrast of the training images to ensure the randomness of the sample and ensure that the training is not affected by irrelevant factors.

For the end-to-end training of the segmentation stage, the number of training epochs is set to 100, and the batch size is set to 8. We use the Adam optimizer to iteratively update the neural network weights based on the training data, the loss function selects the cross entropy, and a checkpoint is used in the training process to get the best model. In the detection stage, we used the stochastic gradient descent for back propagation and weight decay, and the number of training steps is set to 50k. Due to memory issues with the GPU, the batch size is set to 1. The learning rate is set to 0.00125 in the first 25k steps, 0.000125 from steps 25k to 40k, and it drops to 0.0000125 after 40k steps. The weight is set to 0.0001. The optimizer is the SGD, the MOMENTUM is set to 0.9, and Nesterov gradient acceleration is used. A mass is considered to be detected if the overlap ratio between the bounding box of the candidate region and ground truth is 0.3, which is similar to other works in the field [32–34]. We use the transfer learning method to initialize the parameters of the deep learning models, and the Microsoft COCO dataset [38] is utilized to pretrain the deep model. All of these experiments are trained on a PC with the following specifications: Intel® Core i7-9700K CPU with a 3.360 GHz clock speed or frequency, 32 GB of RAM, an NVIDIA GeForce GTX 1080 GPU, and the Ubuntu 18.04 operating system. The deep segmentation models are implemented utilizing the Theano and Keras deep learning libraries, and the detection models are implemented under the TensorFlow environment.

### 3.2. Evaluation Metrics and Results

#### 3.2.1. Evaluation of the Lung Segmentation Stage

To evaluate the performance of the segmentation method, we use the two common evaluation indicators of the Dice coefficient and Intersection over Union (IoU) to evaluate the medical image segmentation performance of the segmentation stage in our proposed system. The Dice indicator is the ratio of the intersecting area to the total area between the ground truth images (G) and the segmented images (O). It can be computed by using equation (1), and the IoU measure can be calculated using equation (2):

(1) $$\begin{array}{c} \text{Dice}=\frac{2\left|G\cap^{}O\right|}{\left|G\right|+\left|O\right|}, \end{array}$$

(2) $$\begin{array}{c} \text{IoU}=\frac{\left|G\cap O\right|}{\left|G\cup O\right|}. \end{array}$$

In the segmentation stage, considering the capacity of the GPU memory, we compared the segmentation experiments with different resolution images as input. As shown in Table 1, we found the result that resolution has little effect on the segmentation of the lung region, which can be ignored. In consideration of the model's computational cost and segmentation performance, we selected a more appropriate 256 × 256 pixels size setting for the image so that the results are comparable with the state-of-the-art approaches during the segmentation stage.

**Figure 6:**
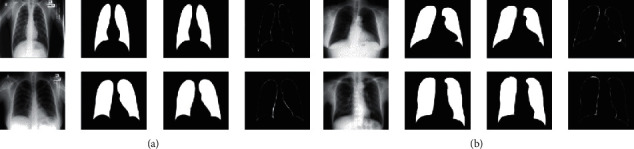
Segmentation results: (a) JSRT and (b) Montgomery. The columns are the original image, segmented mask, ground truth, and difference of the segmented mask from the ground truth from left to right.

In the segmentation stage, as shown in Figure 6, our segmentation model can completely segment the lung region, especially the lung region where the tuberculosis lesions are located. We have achieved fairly good segmentation results that accurately define the region-of-interest and can be used by the detection stage of the computer-aided system. Compared with the other methods in Table 2, our method has achieved better segmentation results so far.

#### 3.2.2. Evaluation of the Multicategory Tuberculosis Lesion Detection Stage

We use the Mean Average Precision (MAP) that is commonly used to assess multiobjective detection models to evaluate our model in this paper. First, we calculated the IoU of the prediction box and ground truth. We specified that the prediction box whose intersection ratio exceeded 0.3 was the correct prediction. Then, we calculate the number of correct detections for each category in each graph, divide that by the total number of targets of this category in the graph, and obtain the accuracy of this category in the graph, as shown in the following equation:

(3) $$\begin{array}{c} {\text{Precision}}_{C}=\frac{N{\left(\text{True Positives}\right)}_{C}}{N{\left(\text{Total Objects}\right)}_{C}}. \end{array}$$

Then, the accuracy of each category is calculated for all test images, and their average is calculated to obtain the average accuracy of this class, as shown in the following equation:

(4) $$\begin{array}{c} \mathrm{Average}\;{\text{Precision}}_{C}=\frac{\sum^{}{\text{Precision}}_{C}}{N{\left(\text{Total Images}\right)}_{C}}. \end{array}$$

Finally, we integrate all the classes in our dataset and average the average precision of each class to get the mean average precision, as shown in the following equation:

(5) $$\begin{array}{c} \mathrm{Mean}\;\mathrm{Average}\;{\text{Precision}}_{C}=\frac{\sum^{}{\text{AveragePrecision}}_{C}}{N\left(\text{Classes}\right)}. \end{array}$$

**Figure 7:**
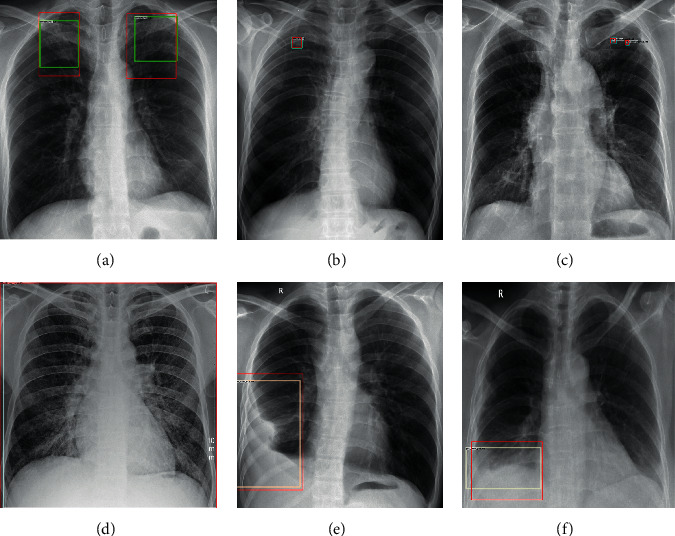
The red boxes are the ground truths, and the green, blue, and white boxes represent the results of our proposed method. The lesion categories: (a) exudation, (b) nodules, (c) calcification, (d) miliary tuberculosis, (e) encapsulated pleural effusion, and (f) free pleural effusion.

We compared the detection results of the Faster RCNN model and the Faster RCNN model with the added original feature pyramid structure. The following Figure 7 shows the detection results of our model. We can see that our methods can well detect the lesions with different sizes and distributions. As seen from Table 3, we show that the computer-aided system can effectively detect tuberculosis lesions and effectively solve the challenges of tuberculosis lesions with different distributions and sizes, especially for small lesions such as nodules, exudation, etc. Our system can significantly improve the detection of small targets. Due to the unevenness of the detection data, the detection of large-area lesions slightly declines, but the overall detection of multiple categories of tuberculosis lesions has greatly improved.

#### 3.2.3. Evaluation of the Proposed Computer-Aided System for Whole Chest X-Rays

To compare our method with existing tuberculosis computer-aided systems, a classification rule based on the tuberculosis detection model is proposed to verify the effectiveness of the proposed system. We tested the proposed strategy for classifying chest X-rays using the tuberculosis detection model on the local collected dataset and the two publicly available tuberculosis datasets [26], Montgomery dataset and Shenzhen dataset. The corresponding lesion area and the probability of the lesion being the disease are calculated for each chest piece. The highest probability of lesions in each chest radiograph was selected as the final lesion probability of the chest radiograph. The threshold range was set to (0, 1), and the interval was 0.1 for the classification analysis. Here, tuberculosis is regarded as the positive class, and no tuberculosis is regarded as the negative class. When a normal chest radiograph is determined to have a tuberculosis lesion, if the probability of the lesion is greater than the currently set threshold, then it is judged as a false positive (FP); and if it is less than the threshold, it is classified as a true negative (TN). Similarly, when a chest radiograph with tuberculosis is determined to have a tuberculosis lesion, if the probability of the lesion is greater than the currently set threshold, it is judged as a true positive (TP); and if it is less than the threshold, it is a false negative (FN). Each image is classified according to the above rules. When the threshold is set to 0.6, the classification accuracy, specificity, and sensitivity of the proposed computer-aided system are calculated separately using equations (6)–(8), respectively:

(6) $$\begin{array}{c} \text{Accuracy}=\frac{\text{TP}+\text{TN}}{\text{TP}+\text{TN}+\text{FP}+\text{FN}}, \end{array}$$

(7) $$\begin{array}{c} \text{Specificity}=\frac{\text{TN}}{\text{TN}+\text{FP}}, \end{array}$$

(8) $$\begin{array}{c} \text{Sensitivity}=\frac{\text{TP}}{\text{TP}+\text{FN}}. \end{array}$$

The classification results are also evaluated using the receiver operating characteristic curve (ROC), and we also obtain the area under the ROC curve (AUC). In Table 4, compared with other methods, our method has achieved the best results so far, especially on the Montgomery dataset, which has improved significantly. Due to the lower image resolution of the Shenzhen dataset, the improvement of the system on the Shenzhen dataset is slightly lower than that for the Montgomery dataset. In addition to the good results obtained on the two public datasets [26], the proposed system is evaluated on the local dataset, and its performance reaches the best results of AUC = 0.993 and ACC = 0.974 (as shown in Table 4). The system can achieve effective auxiliary diagnosis of tuberculosis diseases. Overall, our proposed system has better performance in the diagnosis of tuberculosis.

**Figure 8:**
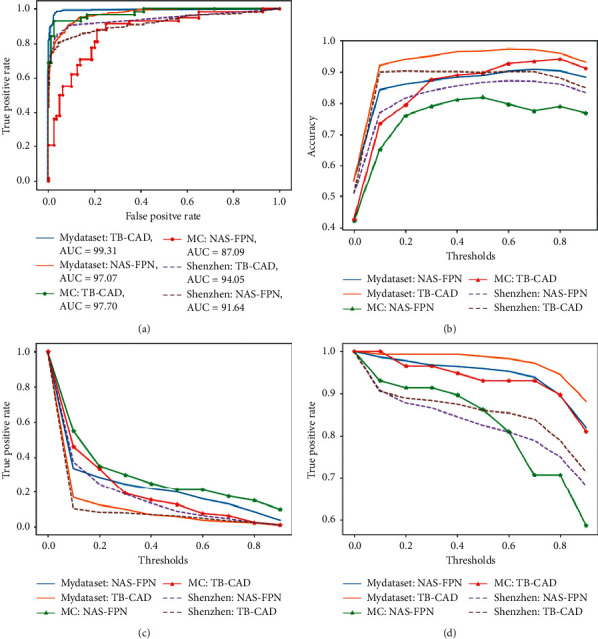
The Performance of the proposed computer-aided system on whole chest X-rays on local dataset (MyDataset) and two public datasets, the Montgomery dataset (MC), and the Shenzhen dataset (ShenZhen). (a) ROC, (b) accuracy, (c) false positive rate, and (d) true positive rate.

We select the detection model trained by the NAS-FPN network [34] as the baseline model. We use the classification rule on the baseline model for further analysis. We plot the accuracy, ROC curve, ture positive rate, and false positive rate when the classification threshold is changed within the range of [0, 1]. As shown in the following Figure 8, our system is a significant improvement over baseline model on the two public datasets [26] and the local dataset, and our proposed system can effectively reduce the false positive rate and improve the sensitivity and accuracy.

## 4. Conclusions

This paper proposes a computer-aided system, which is based on deep learning, for the detection of multiple categories of tuberculosis lesions in radiographs. For tuberculosis, the proposed computer-aided system can automatically segment the lung area and then detect the multiple categories of tuberculosis lesions in the segmented lung X-rays to achieve the auxiliary analysis and diagnosis of tuberculosis. Therefore, it can play a vital role in forming the second opinion of a radiologist. In the segmentation stage, the system achieves higher segmentation performance on the two public datasets [26, 35] than current methods. In addition, because of the segmentation capability of the deep model that was used, the segmented image is used in the detection stage, and the computer-aided system can achieve better detection results. For the detection of tuberculosis, this system is different from the previous method that conducts classification using the whole CXR. It is focusing on the regions with tuberculosis lesions, incorporates the learning scalable pyramid structure into the Faster RCNN, and applies reinforcement learning in the trained lesion detection model, which forms the proposed multicategory tuberculosis lesion detection method. Multicategory tuberculosis detection methods can effectively detect tuberculosis lesions with different sizes, locations, and categories, especially for small targets such as lung nodules. The proposed deep learning computer-aided system achieves higher performance on the two public datasets [26] than current methods. Compared with the baseline model [34], the system effectively reduces the false positive rate and improves the sensitivity. The proposed deep learning computer-aided system integrates trained segmentation and detection models and can be implemented on ordinary computers. The system can be used in clinical applications to assist radiologists in diagnosis and public health providers in screening for tuberculosis in areas where tuberculosis is endemic. For low- and middle-income countries with high incidence of tuberculosis, this system is very practical and economically useful for tuberculosis detection.

## Figures and Tables

**Table 1:** Precision of the proposed method using the Dice and IoU on various resolutions.

Resolution	Dataset	Dice	IoU
256 × 256	JSRT	0.980	0.961
	Montgomery	0.978	0.957

512 × 512	JSRT	0.980	0.962
	Montgomery	0.979	0.959

1024 × 1024	JSRT	0.980	0.960
	Montgomery	0.977	0.955

**Table 2:** Performance of the proposed computer-aided system in the segmentation stage.

Method	Year	Dataset	Dice	IoU
Peng et al. [31]	2019	JSRT	0.965	0.932
Johnatan Carvalho Souza [30]	2019	Montgomery	0.936	0.881
Mittal et al. [29]	2018	JSRT + Montgomery	—	0.951
Rahul Hooda et al. [28]	2018	JSRT	0.958	0.917
Novikov et al. [27]	2018	JSRT	—	0.95
Ours method	2019	JSRT	**0.980**	**0.961**
		Montgomery	**0.978**	**0.957**

**Table 3:** Performance of the proposed computer-aided system in the detection stage.

Method	Mean average precision	Average precision					
		Exudation	Calcification	Nodule	Miliary tuberculosis	Free pleural effusion	Encapsulated pleural effusion
Faster RCNN	22.66	41.49	5.52	0.37	40.57	45.65	2.36
FPN	50.96	52.89	**42.84**	17.50	**87.72**	52.67	52.13
Our method	**53.74**	**55.54**	41.82	**22.73**	84.62	**64.92**	**52.79**

**Table 4:** Performance of the proposed computer-aided system on whole chest X-rays.

Method	Year	Dataset	AUC	Accuracy	Sensitivity	Speciﬁcity
Thi Kieu Khanh Ho et al. [19]	2019	Shenzhen	0.914	—	—	—
		Montgomery	0.939	—	—	—
Satyavratan et al. [12]	2019	Montgomery	0.94	0.878	0.877	0.859

Sivaramakrishnan et al. [18]	2018	Shenzhen	0.926	0.855	—	—
		Montgomery	0.833	0.758	—	—
Mohammad et al. [21]	2017	Shenzhen	0.940	0.900	0.88	0.92

Lopes et al. [17]	2017	Shenzhen	0.926	0.847	—	—
		Montgomery	0.926	0.826	—	—

Sangheum Hwang et al. [20]	2016	Shenzhen	0.926	0.837	—	—
		Montgomery	0.884	0.674	—	—

Our method	2019	Shenzhen	**0.941**	**0.902**	**0.854**	**0.951**
		Montgomery	**0.977**	**0.926**	**0.931**	**0.923**
		Local	**0.993**	**0.974**	**0.983**	**0.962**

## Data Availability

The chest radiograph data used to support the findings of this study have not been made available because of the patients' privacy. The chest radiograph data used to support the findings of this study are available from the corresponding author upon request.
